# Impaired reasoning and problem-solving in individuals with language impairment due to aphasia or language delay

**DOI:** 10.3389/fpsyg.2015.01523

**Published:** 2015-10-26

**Authors:** Juliana V. Baldo, Selvi R. Paulraj, Brian C. Curran, Nina F. Dronkers

**Affiliations:** ^1^VA Northern California Health Care System, Martinez, CAUSA; ^2^Palo Alto University, Palo Alto, CAUSA; ^3^Center for Mind and Brain, University of California, DavisDavis, CA, USA; ^4^National Research University Higher School of Economics, MoscowRussian Federation

**Keywords:** reasoning, problem-solving, aphasia, language delay, deafness, thought, language, inner speech

## Abstract

The precise nature of the relationship between language and thought is an intriguing and challenging area of inquiry for scientists across many disciplines. In the realm of neuropsychology, research has investigated the inter-dependence of language and thought by testing individuals with compromised language abilities and observing whether performance in other cognitive domains is diminished. One group of such individuals is patients with aphasia who have an impairment in speech and language arising from a brain injury, such as a stroke. Our previous research has shown that the degree of language impairment in these individuals is strongly associated with the degree of impairment on complex reasoning tasks, such as the Wisconsin Card Sorting Task (WCST) and Raven’s Matrices. In the current study, we present new data from a large group of individuals with aphasia that show a dissociation in performance between putatively non-verbal tasks on the Wechsler Adult Intelligence Scale (WAIS) that require differing degrees of reasoning (Picture Completion vs. Picture Arrangement tasks). We also present an update and replication of our previous findings with the WCST showing that individuals with the most profound core language deficits (i.e., impaired comprehension and disordered language output) are particularly impaired on problem-solving tasks. In the second part of the paper, we present findings from a neurologically intact individual known as “Chelsea” who was not exposed to language due to an unaddressed hearing loss that was present since birth. At the age of 32, she was fitted with hearing aids and exposed to spoken and signed language for the first time, but she was only able to acquire a limited language capacity. Chelsea was tested on a series of standardized neuropsychological measures, including reasoning and problem-solving tasks. She was able to perform well on a number of visuospatial tasks but was disproportionately impaired on tasks that required reasoning, such as Raven’s Matrices and the WAIS Picture Arrangement task. Together, these findings suggest that language supports complex reasoning, possibly due to the facilitative role of verbal working memory and inner speech in higher mental processes.

## Introduction

To what extent is thought dependent on language? This question has been pondered by philosophers and scientists alike for millenia. When asked about the nature of thinking, Socrates stated: “The soul when thinking appears to me to be just talking" (Jowett, 1892, p. 252). Many individuals echo this same subjective experience of an internal dialog that often accompanies their thoughts (Hurlburt, 1990; Hurlburt and Heavey, 2001; Carruthers, 2002), but how can we objectively study the relationship between language and thought? A number of efforts to address this question experimentally have made use of data from a range of sources, including animals, young children, healthy adults, and language-impaired individuals (Watson, 1920, 1924; Piaget, 1967; Kertesz and McCabe, 1975; Vygotsky, 1978; Hjelmquist, 1989; Hurlburt, 1990; Halford et al., 1998; Hermer-Vazquez et al., 1999; Kinsbourne, 2000; Varley and Siegal, 2000; Kuczaj and Hendry, 2003; Wheeler, 2004; Machery, 2005; Clark, 2006; Penn et al., 2008; Carpendale et al., 2009; for a review, see Perrone-Bertolotti et al., 2014). In the current paper, we focus on the role that language plays in reasoning and problem-solving in particular. First, we review prior work in this area from a range of sources and then describe our current research focused on studying the relationship between language and reasoning in individuals with varying degrees of language impairment.

Evidence from a number of animal studies clearly demonstrate remarkable reasoning and problem-solving abilities in non-human species (Blaisdell et al., 2006; Taylor et al., 2009; Smirnova et al., 2015), but it is argued that such abilities have reached a higher level in humans (Premack, 1983, 2007; O’Brien and Opie, 2002; Penn et al., 2008). Since abstract, symbolic language reaches its apex in humans as well, it has been suggested that these abilities are causally related, that is, our language system facilitates logical reasoning in some way (Sokolov, 1968/1972; Premack, 1983; Carruthers, 2002; O’Brien and Opie, 2002; Bermudez, 2003; Gentner, 2003; Kuczaj and Hendry, 2003; Goel and Dolan, 2004). Interestingly, it has been shown that chimpanzees who receive language-training have superior reasoning and problem-solving skills compared to language-naïve chimpanzees (Premack, 1983), supporting the notion that representational language facilitates advanced reasoning.

Data from children also suggest that language plays an important role in thought and reasoning (Behrend et al., 1989; Gentner and Loewenstein, 2002; Gentner, 2003; Loewenstein and Gentner, 2005; Lidstone et al., 2012). Vygotsky (1978, 2012) argued that young children first use overt speech to work through problems in conjunction with elders, then learn to speak to themselves privately while working through problems on their own, and eventually internalize that overt speech into private, covert speech while problem-solving. Piaget also believed that children’s private speech supported thinking and was related to the development of reasoning ability (Piaget, 1926, 1967). Young children verbalize overtly when performing cognitively demanding tasks, but by 14–17 years of age, children report using an inner speech strategy (Winsler and Naglieri, 2003). Experimental evidence for these ideas comes from studies showing that problem-solving performance in children is associated with the use of private speech: Fernyhough and Fradley (2005) showed that the use of self-regulating statements in children correlated with performance on the Tower of London puzzle task (see also Winsler et al., 2009). Similarly, it has been shown that children exhibit increased self-directed and private speech when performing a difficult task and that children who exhibit more self-directed speech are better able to solve problems (Berk and Garvin, 1984; Berk, 1986; Behrend et al., 1989; Winsler et al., 1997). Finally, others such as Hermer-Vazquez et al. (1999) have shown that children’s ability to perform a problem-solving task involving spatial orientation is related to their level of language competence (but see Learmonth et al., 2008).

Other work has approached the study of the relationship between language and reasoning by assessing cognitive functioning in individuals with varying degrees of language impairment (Kinsbourne, 2000; Varley and Siegal, 2000). A series of findings have shown that individuals with aphasia (an impairment in language due to brain injury) show deficits in reasoning and problem-solving (e.g., Weinstein and Teuber, 1957; Piercy, 1964; De Renzi et al., 1966; Archibald et al., 1967; Basso et al., 1973; Edwards et al., 1976; Borod et al., 1982; Larrabee, 1986; Hjelmquist, 1989; Hamsher, 1991; Baldo et al., 2005, 2010; but see Kinsbourne and Warrington, 1963; Basso et al., 1973). Moreover, a large number of these studies have shown that the degree of aphasia severity is correlated with the level of cognitive impairment (De Renzi et al., 1966; Archibald et al., 1967; Edwards et al., 1976; Borod et al., 1982; Larrabee, 1986; Vilkki, 1988; Baldo et al., 2005, 2010; but see Basso et al., 1973; Helm-Estabrooks, 2002). In particular, individuals with severe comprehension deficits such as those with Wernicke’s aphasia appear to be especially impaired on problem-solving and reasoning tasks (Kertesz and McCabe, 1975; Hjelmquist, 1989; Baldo et al., 2005), a finding not simply explained by a failure to understand task instructions.

In our work, we have examined the role of language in reasoning by comparing large groups of stroke patients with and without aphasia on standardized tests of non-verbal reasoning and problem-solving. Such tests require the ability to recognize/represent a problem, use available information to test possible solutions, and monitor the veracity of those solutions. By “non-verbal,” we refer to the fact that these tasks do not require a spoken response and have no/minimal language comprehension requirements. For example, Baldo et al. (2005) tested 41 right and left hemisphere stroke patients with a wide range of aphasia severity on the Wisconsin Card Sorting Test (WCST; Heaton et al., 1993) and showed that problem-solving performance was significantly related to the degree of patients’ language impairment. Interestingly, performance was most strongly related to patients’ comprehension scores in particular, suggesting that core language processes are most important for successful problem-solving. In an effort to establish discriminant validity (i.e., to show that the relationship between language scores and performance on the WCST was not simply a matter of overall cognitive impairment), we also showed that there was no relationship between patients’ language scores and performance on Block Design, a non-verbal test of visuospatial functioning that is minimally dependent on reasoning. This dissociation reinforces the idea that individuals with aphasia, particularly those with core language impairments, have difficulty on tasks involving reasoning that are not explained by a general cognitive impairment.

**FIGURE 1:**
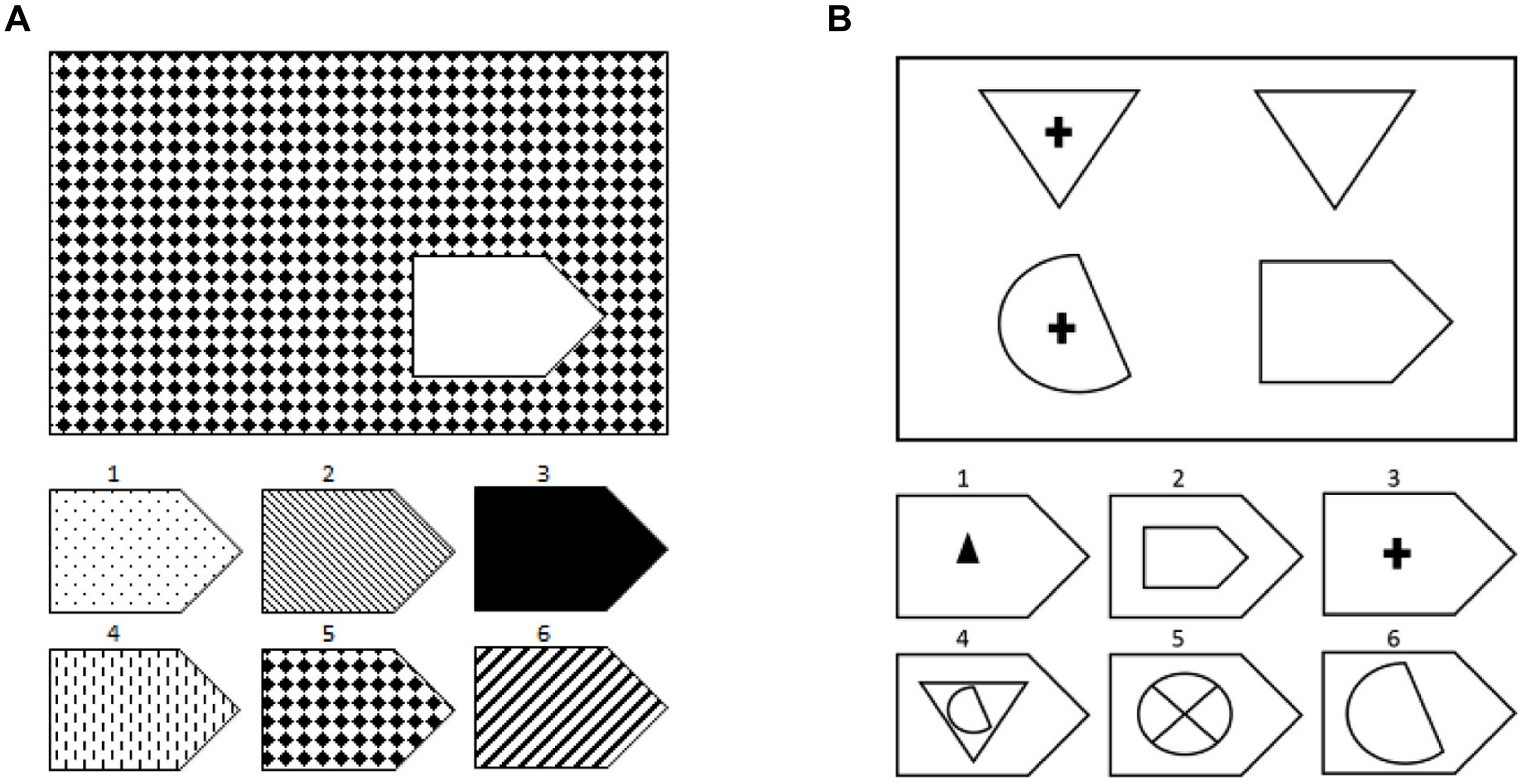
**Examples of the types of problems on Raven’s Matrices: **(A)** visual-pattern completion and **(B)** relational reasoning.** These are not actual items from the test due to copyright issues and to maintain test security.

Similarly, we have shown that aphasic individuals also exhibit poor performance on another test of non-verbal reasoning, Raven’s Coloured Progressive Matrices (Baldo et al., 2010). Importantly, there was a significant interaction in performance on this test such that individuals with aphasia were disproportionately impaired on Raven’s items that required relational reasoning relative to those items that only required visual-pattern completion (see **Figure 1** for examples; Bunge et al., 2005; Crone et al., 2009). Again, the specificity of these findings bolster the conclusion that decrements in reasoning in particular are associated with language impairment following stroke, rather than such deficits being part of a more general cognitive impairment.

In the current paper, we sought to extend our previous findings by testing the relationship between aphasia severity and reasoning using a series of putatively non-verbal tasks from a commonly administered instrument, the Wechsler Adult Intelligence Scale (WAIS; Experiment 1A). We also sought to replicate and extend our previous findings with the WCST in a larger and more homogeneous patient sample that included left hemisphere-injured patients only (Experiment 1B). Rather than focus solely on aphasia subtypes, which can be problematic due to the multi-dimensional nature of these syndromes (Caramazza and McCloskey, 1988; Coltheart, 2004), we also assessed the relationship between reasoning performance and specific language sub-processes (e.g., auditory comprehension, repetition). In the second part of this paper, we describe findings from a unique individual whose language impairment derives not from aphasia but from the fact that she was not exposed to language until the age of 32 due to an unaddressed hearing loss (Experiment 2). Together, these data provide further insights into the close relationship between language and reasoning.

## Experiment 1A: Reasoning Performance in Aphasic Individuals on the WAIS

In a further effort to understand the role of language in reasoning, we conducted an analysis of aphasic patients’ performance on the Picture Completion and Picture Arrangement subtests of the WAIS-R and WAIS-III (Wechsler, 1981, 1997). These standardized tasks were chosen for comparison because they both require visual perception and attention with no/minimal language or motor requirements, but differ with respect to the amount of reasoning required (Ryan and Paolo, 2001; Tulsky et al., 2003). A previous study (Varley, 1998) used the Picture Arrangement task as a measure of causal reasoning in aphasia and reported that one of the two aphasic individuals tested showed impaired performance; however, there was concern that visual impairments could have contributed to performance. Given these equivocal results, it was of interest to compare performance on the Picture Arrangement task to performance on the Picture Completion task, which also involves visual perception and attention but a smaller reasoning component. Also, we analyzed performance from a large sample of well-characterized left hemisphere patients with a range of language disturbance and no known visual disturbance. Our prediction was that aphasic individuals would be disproportionately impaired on the more reasoning-intensive Picture Arrangement task, relative to Picture Completion and that patients’ comprehension scores would be most strongly correlated with performance.

### Participants

A retrospective analysis was performed on data from 60 individuals (17 female) in our database who met strict inclusion/exclusion criteria: history of a single left hemisphere stroke, at least 6 months post-stroke (to ensure that behaviors had stabilized), native English-speaking (by age 5), right-handed, 8^th^ grade education or higher, and no prior neurologic or severe psychiatric history. The determination of language impairment was made with the Western Aphasia Battery (WAB; Kertesz, 1982, 2006), which also provides scores for language sub-processes (fluency, comprehension, naming, etc.) as well as an aphasia subtype diagnosis (i.e., Broca’s, Wernicke’s, conduction aphasia, etc.). Individuals that score above the cut-off for normal language on the WAB (93.7 out of 100 points possible) are considered non-aphasic according to the WAB manual and norms. Based on this cut-off, our sample included 37 individuals with aphasia and 23 non-aphasic individuals. The aphasic individuals included 17 individuals with anomic aphasia, nine with Broca’s aphasia, five with conduction aphasia, five with Wernicke’s aphasia, and one individual who was unclassifiable. Patients’ mean age ±*SD* was 61.7 ± 11.0 years for the aphasic individuals and 59.6 ± 10.9 years for non-aphasic individuals; mean education was 14.6 ± 2.3 years for the aphasic individuals and 15.8 ± 3.0 years for non-aphasic individuals; mean months post-stroke was 44.4 ± 46.9 months for aphasic individuals and 41.7 ± 48.3 months for non-aphasic individuals; and mean lesion volume was 136.1cc ± 71.6 for aphasic individuals and 31.5cc ± 29.0 for non-aphasic individuals. Finally, the aphasic group included eight women and the non-aphasic group included six women.

### Materials and Procedures

Participants were administered the Picture Completion and Picture Arrangement subtests from the WAIS-R or WAIS-III. The Picture Completion task requires examinees to point to something missing in a series of drawings of increasing difficulty (e.g., a number missing from a keypad). The Picture Arrangement task requires examinees to rearrange a series of pictures so that they tell a story, like the tiles in a comic strip (e.g., a series of pictures showing different stages of people cooking a meal). While both tasks require visuo-spatial perception and attention, the Picture Arrangement task puts a greater burden on reasoning ability (Varley, 1998; Tulsky et al., 2003). The tasks were administered and scored in the standard manner according to the WAIS manual. Because the data were collected over a period of years, some participants were administered the Picture Completion and Picture Arrangement subtests from the WAIS-R and others, the WAIS-III. In order to combine data from the WAIS-R and WAIS-III, scores were adjusted according to Gläscher et al. (2009), which involved converting the WAIS-R raw scores to WAIS-III raw scores by adding the mean difference to each subtest (-0.4 for Picture Completion and -0.6 for Picture Arrangement). Last, we also analyzed data from the Benton Face Recognition Task (Benton et al., 1983) for these 60 individuals. The Benton Face Recognition Task is a visuo-perceptual task in which examinees are asked to point to which of six faces on the bottom of the page is the same person as the face on top. Despite being called a *face recognition* task, it is simply a face-matching task involving non-famous faces with no delay.

An analysis of covariance (ANCOVA) was used to compare aphasic and non-aphasic individuals’ raw scores on the different tasks with age, years of education, months post-stroke, and lesion volume included as covariates. Partial correlation coefficients (two-tailed) were computed to relate reasoning performance to WAB subtest scores for speech fluency, object naming, repetition, and auditory comprehension, with the same nuisance factors as control variables. The WAB fluency score is a rating from 0 to 10 of an individual’s spontaneous speech based on fluency of speech, grammatical competence, and paraphasic errors; the WAB naming subtest involves naming a series of 20 physically presented items (e.g., ball, cup); the WAB repetition subtest requires examinees to repeat 15 items that include single words, phrases, and sentences; and the WAB auditory comprehension score is based on yes/no questions, single-word recognition (both physical object-word matching and picture-word matching), and sequential commands.

### Results

**FIGURE 2:**
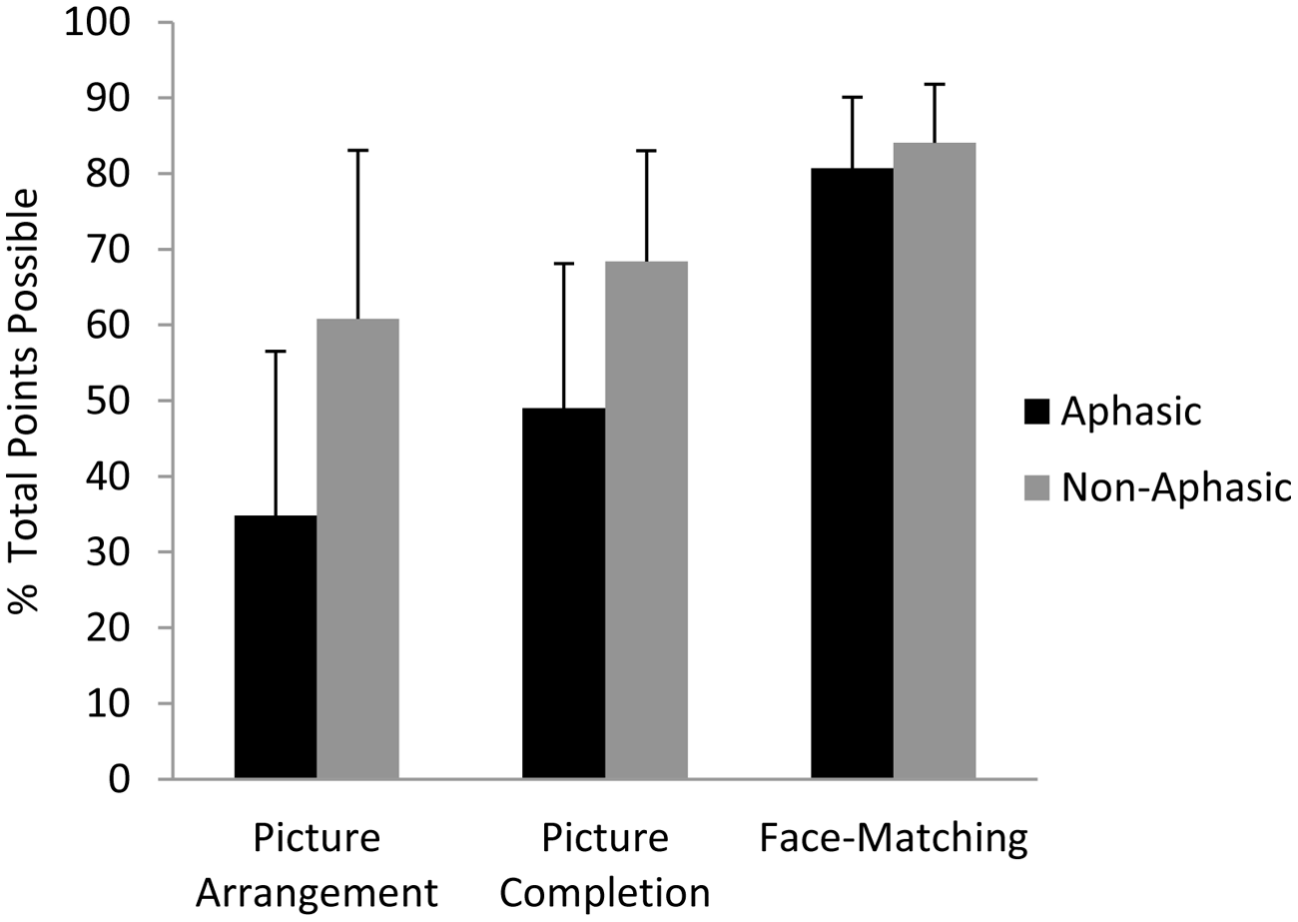
**Performance of aphasic and non-aphasic stroke patients on the WAIS Picture Completion and Picture Arrangement subtests, and the Benton Face Recognition (Matching) Task.** The raw data (number of points attained on each task) were converted to a percentage of total points possible for each task in order to compare the results across tasks. Standard deviation bars are shown.

As predicted, the ANCOVA showed that aphasic individuals performed significantly poorer than non-aphasic individuals on the Picture Arrangement task, *F*(1,54) = 6.25, *p* = 0.04 (*M* ±*SD* = 34.8 ± 21.7% vs. 60.8 ± 22.3%, respectively), but the groups did not differ statistically on the Picture Completion task (49.0 ± 19.1% vs. 68.5 ± 14.6%, respectively), *F*(1,54) = 1.51, *p* = 0.24 (see **Figure 2**), controlling for age, education, months post-stroke, and lesion volume. Again, these results suggest that language impairment is related to reduced performance on tasks that place a greater demand on reasoning ability, even when that task does not require any overt language production. As in our previous work, the most severely language-compromised individuals in the sample, those with Wernicke’s aphasia, had the numerically lowest performance on the Picture Arrangement (reasoning) task. In keeping with this result and similar to our previous findings, partial correlations revealed significant relationships between scores on the Picture Arrangement task and comprehension, *r*(58) = 0.27, *p* = 0.04, as well as repetition, *r*(58) = 0.28, *p* = 0.03, but not with naming or fluency (*p*s > 0.05). The same pattern held true when we repeated the partial correlation analyses with the sub-sample of aphasic individuals only, with comprehension and repetition alone showing marginally significant correlations of 0.33 (*p* = 0.07) and 0.32 (*p* = 0.08), respectively. With respect to normative cut-offs based on age-adjusted norms provided by the WAIS manual, 13.5% of the aphasic individuals performed in the significantly impaired range on the Picture Arrangement task (age-adjusted scale score of 4 or lower) but only a single individual in the non-aphasic subgroup. On the Picture Completion task, 18.9% of aphasic individuals performed in the significantly impaired range and none of the non-aphasic individuals.

As can be seen in **Figure 2**, aphasic individuals exhibited numerically (though not statistically) poorer performance than non-aphasic individuals on the Picture Completion task as well. For this reason, we additionally analyzed performance from these same 60 individuals on another standardized test of visuospatial functioning that does not involve reasoning, the Benton Face Recognition Task. On this more purely visual-perceptual task, aphasic and non-aphasic individuals performed comparably as revealed by ANCOVA, *F*(1,54) = 0.72, *p* = 0.40 (*M* ±*SD* = 81 ± 9.4% vs. 84 ± 7.7%, respectively; see **Figure 2**).

These findings are consistent with the notion that language facilitates reasoning. Specific correlations between problem-solving performance and comprehension and repetition further suggest that core language processes (as opposed to output processes such as fluency and naming) are most strongly related to performance. It should be emphasized that poor performance cannot simply be explained by individuals with severe language impairments misunderstanding the task, as they are able to demonstrate their understanding of task instructions in the initial trials, before it becomes more difficult. In the Discussion, we explore potential explanations of the observed relationship between compromised core language and impaired reasoning as they relate to the supportive role of inner speech and working memory.

## Experiment 1B: WCST Performance in Individuals with Aphasia

We previously showed that performance on the WCST, a standardized measure of problem-solving and reasoning, was impaired in aphasic individuals and also correlated with a number of critical language variables (Baldo et al., 2005). However, our paper included a relatively small sample and a heterogeneous group of both right and left hemisphere patients. Therefore, we sought to replicate our previous findings in a larger sample of patients whose lesions were restricted to the left hemisphere, in order to confirm our previous findings of a relationship between language impairment and problem-solving performance.

### Participants

A retrospective data analysis was conducted on data from 81 chronic left hemisphere stroke patients from our database (23 female) who met the same strict inclusion/exclusion criteria described above in Experiment 1A. Seventeen of these patients were also included in Baldo et al. (2005). Based on the WAB, 35 patients were non-aphasic (i.e., scored within normal limits) and 46 patients were aphasic. This latter group included 20 individuals with anomic aphasia, 12 with Broca’s aphasia, seven with conduction aphasia, two with transcortical sensory aphasia, and five with Wernicke’s aphasia. Patients’ mean age ±*SD* was 60.6 ± 11.6 years for the aphasic individuals and 60.5 ± 10.3 years for non-aphasic individuals; mean education was 14.3 ± 2.7 years for the aphasic individuals and 15.3 ± 2.8 years for non-aphasic individuals; mean months post-stroke was 48.5 ± 50.5 months for aphasic individuals and 44.7 ± 53.2 months for non-aphasic individuals; and mean lesion volume was 126.2cc ± 67.1 in aphasic individuals and 35.1cc ± 43.6 in non-aphasic individuals. Finally, the aphasic group included 12 women and the non-aphasic group included 11 women.

### Materials and Procedures

The WCST requires examinees to match test cards with different arrays of 1–4 items (e.g., two triangles) drawn in different colors to one of four key cards with similar arrays. Participants are not told how to match the test cards to the key cards but are provided with feedback from the examiner (*correct* or *incorrect*) after each move. Unbeknownst to the examinee, the examiner repeatedly changes the sorting category after a set number of trials, and the examinee must recognize this switch and modify their sorting behavior based on the feedback. As an indication of adequate comprehension of task instructions, only a single individual with Wernicke’s aphasia was unable to sort at least 1 category.

Aphasic and non-aphasic individuals’ raw scores were compared with an ANCOVA using age, years of education, months post-stroke, and lesion volume as covariates as above. In addition, individuals’ praxis subtest scores from the WAB were included as an extra covariate to ensure that poor performance was not related to ideomotor apraxia (Basso et al., 1981; a subset of 10 individuals in the sample scored below 80% correct on the praxis subtest). Partial correlation coefficients (two-tailed) were computed with the same nuisance factors, in order to test the relationship between problem-solving performance on the WCST and language processes including speech fluency, naming, repetition, and auditory comprehension.

### Results

Confirming our 2005 findings, the left hemisphere stroke patients with aphasia performed poorly on the WCST, completing an average of just 2.6 out of 6 possible category sorts (*SD* = 2.1), compared to non-aphasic left hemisphere stroke patients who completed an average of 4.4 out of 6 category sorts (*SD* = 1.9). An ANCOVA confirmed that this difference was significant, *F*(1,64) = 4.16, *p* = 0.04, correcting for age, education, praxis, and months post-onset. The size of patients’ lesions, another potential confound, was available for 79 of the patients and did not change the results when included as an additional covariate.

**FIGURE 3:**
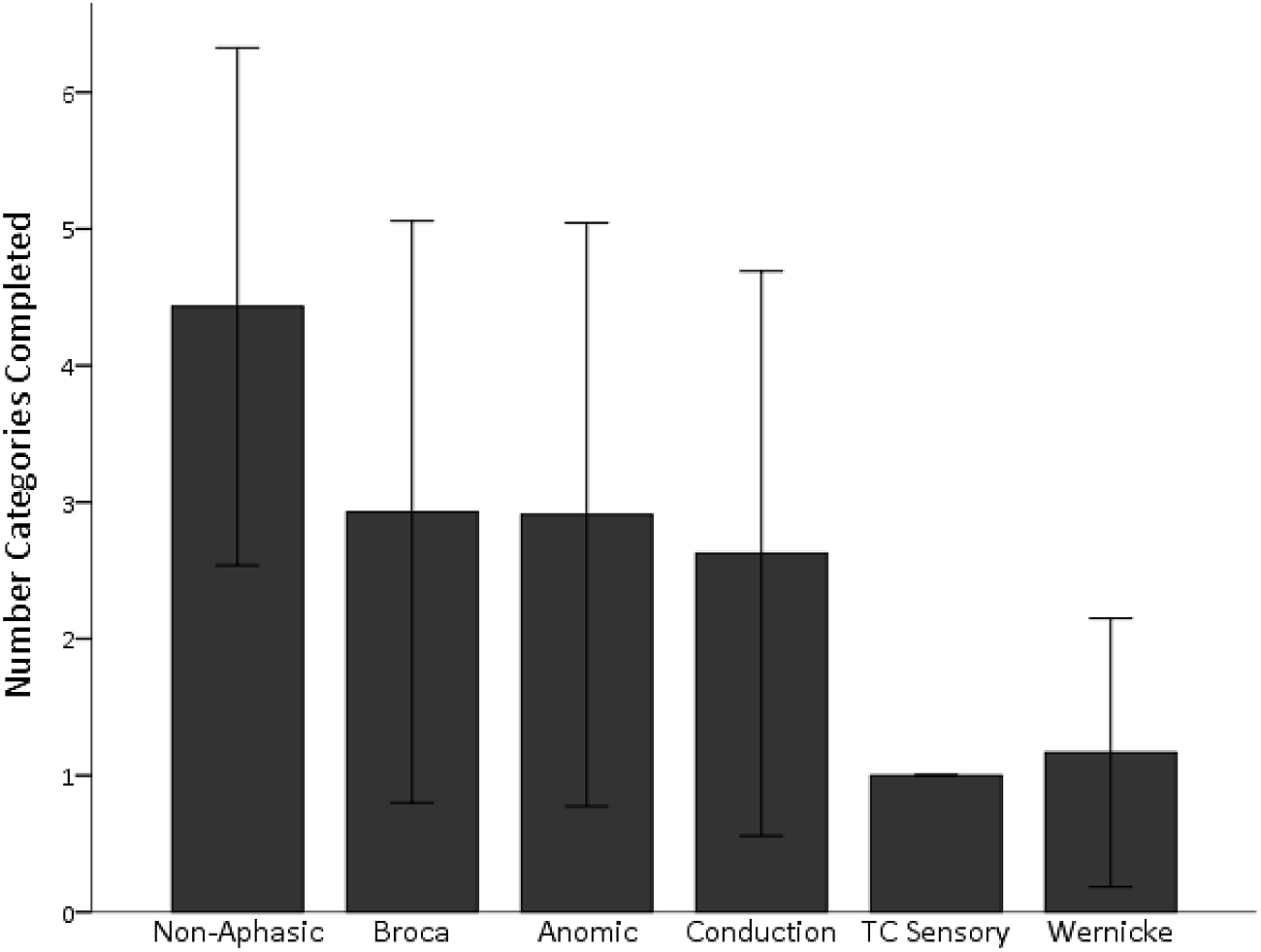
**Wisconsin Card Sorting Task (WCST) performance is shown for the number of categories completed, based on aphasia subtype.** Individuals with severe comprehension deficits (Wernicke’s and TC Sensory aphasia) sorted the fewest number of categories on the WCST. Performance in individuals with milder deficits overlapped with that of non-aphasic individuals. TC Sensory, transcortical sensory aphasia. Standard deviation bars are shown.

As in our previous studies, aphasic individuals with the most severe comprehension deficits, those with Wernicke’s and transcortical sensory aphasia, were particularly impaired and were the only subgroups significantly impaired relative to the non-aphasic group, Dunnett’s T3 (equal variances not assumed), *p*s < 0.001 (see **Figure 3**). As shown, individuals with milder comprehension impairments (i.e., Broca’s, anomic, and conduction aphasia) performed in the moderately impaired range on the WCST task. Since aphasia subtypes can be problematic as they are multi-determined syndromes, we also analyzed WCST performance in relation to specific language subprocesses as measured by the WAB, including speech fluency, auditory comprehension, repetition, and naming. Partial correlations revealed that overall performance on the WCST based on the number of categories sorted was significantly related to auditory comprehension alone, *r*(65) = 0.46, *p* < 0.001. The same was true when the partial correlation analyses were repeated using data from only the aphasic individuals rather than the entire sample: only auditory comprehension significantly correlated with WCST performance, *r*(37) = 0.41, *p* = 0.01.

These new findings on the WCST reinforce our previous work showing that many individuals with aphasia exhibit difficulties on putatively non-verbal problem-solving tasks and thus suggest a relationship between the presence of language deficits and the degree of impairment in problem-solving capacity. Furthermore, individuals with the most severe language impairments (those with transcortical sensory and Wernicke’s aphasia) performed worse overall, and correlational data showed a significant relationship between comprehension scores and problem-solving performance. These latter findings again suggest that core language processes (as opposed to production processes such as fluency) are most significantly related to problem-solving performance.

In short, we have conducted a series of large-scale studies comparing reasoning/problem-solving in aphasic vs. non-aphasic individuals that suggest a supportive role of language in these abilities. Although concerns about potentially confounding factors such as lesion size and overall cognitive impairment were addressed, one could still argue that brain-injured individuals are not the ideal population for addressing the issue of language and cognition. Thus, we now turn to complementary data obtained from a healthy adult with a severely restricted language capacity.

## Experiment 2: Reasoning in a Deaf Individual with Impaired Language

Another way to examine the role that language plays in reasoning and problem-solving is by assessing cognition in healthy individuals with compromised language abilities. This can happen, for example, in deaf individuals who are not exposed to language until they are older (Siegal et al., 2001; Morgan and Kegl, 2006). One of the authors (ND) has worked with such an individual (pseudonym “Chelsea”) who had an untreated, congenital hearing loss and was not exposed to language or any formal education until the age of 32. In Experiment 2, we describe Chelsea’s performance on non-verbal measures of reasoning in comparison to her performance on other cognitively demanding tasks that do not involve reasoning. Data from this unique individual parallel findings in aphasic individuals and offer additional insights into the role that language plays in reasoning and problem-solving.

### Participants

Data were collected from a single case whose pseudonym is “Chelsea,” as well as her parents and two sisters who served as controls. Chelsea was born with a severe to profound sensorineural hearing loss that went unaddressed due to being raised in a rural setting with limited resources (Dronkers, 1987; Glusker, 1987; Glusker et al., 1990; Curtiss, 2014). Her mother had a viral illness while pregnant with Chelsea that is associated with congenital deafness, but a definitive cause of her sensorineural hearing loss was not established. She was raised in a supportive home with her parents and six siblings, and she functioned normally according to family report: carrying out household chores, taking care of younger siblings, etc. As an infant/toddler, she achieved all developmental milestones at a normal rate and at a pace similar to her siblings (e.g., sitting, crawling, standing, walking, etc.), with the exception of language. According to family report, there were no home-signs used to communicate with Chelsea, but rather she relied on pointing, gestures, and miming to indicate her needs. Home visits and videotapes of the family interacting with Chelsea (without researchers present) confirmed the apparent lack of any home-signing system (see Curtiss, 2014).

At the age of 32, Chelsea was evaluated by a number of medical providers and was fitted with bilateral hearing aids that allowed her to hear speech for the first time. She started receiving instruction in both spoken English and Signing Exact English by a licensed speech pathologist. CT and MRI as well as EEG studies conducted at the time were all normal. The neurologist who evaluated her over several sessions reported that she showed no evidence of neurologic disabilities except for a single neurologic sign of mild hyperreflexia on the left side (Glusker et al., 1990 and personal communication).

After being fitted with bilateral hearing aids, Chelsea gradually began acquiring spoken and receptive language, and she achieved a good command of the English lexicon (i.e., production and comprehension of single words; Curtiss, 2014). However, her ability to process syntax, both in production and comprehension, was extremely limited. Not surprisingly, she was difficult to understand and converse with. Examples of Chelsea’s spontaneous speech are provided in **Table 1**. In contrast, she demonstrated relatively preserved pragmatics, including normal body language, prosody, facial expressions, gesture, etc. (Dronkers, 1987; Dronkers et al., 1998; Curtiss, 2014). Curtiss (2014) concluded that Chelsea’s case provides clear evidence that there exists a critical period for acquiring grammar but not for acquiring other aspects of language such as the lexicon, which continues to grow in Chelsea’s case.

**Table 1:** Examples of Chelsea’s spontaneous speech.

They are buy the grapes.
Two boy stand the play yard play.
That my birthday. 34 birthday.
They are is working house.
The frogs are the boy holding.
Working wine the couch the sit. Couch sit.
My birthday. The girl is blowing the [Chelsea] dinner ice cream.


Cognitive performance data from Chelsea’s parents and two sisters are also reported here for comparison. Her mother and father were tested at ages 52 and 62, and had 6th and 9th grade educations, respectively. While not as restricted in their schooling as Chelsea, her parents’ performance offers an informative comparison. Her two sisters were tested at ages 33 and 36, and had 12 and 13 years of education, respectively.

### Materials and Procedures

Chelsea was administered Raven’s Coloured Progressive Matrices and the WAIS-R Performance subtests at several time points from age 32–41. Raven’s Coloured Progressive Matrices includes a series of 36 non-speeded trials in which examinees have to point to 1 of 6 visual patches that best completes a visual pattern or sequence. She was also administered the WAIS-R Performance subtests, which represent the putatively non-verbal portion of the WAIS and are the preferred means of assessing intellectual functioning in hearing-impaired individuals (Braden, 1992). The subtests include Picture Completion, Picture Arrangement, Block Design, Object Assembly, and Digit-Symbol. As described above, the WAIS-R Picture Completion task involves identifying a missing object in a picture, and the Picture Arrangement task involves rearranging a series of pictures so that they tell a story. Block Design requires examinees to rearrange red and white colored cubes to match a pattern printed in a stimulus book, and performance is based on both speed and accuracy. Object Assembly is a series of jigsaw puzzles that begin with very simple ones and get progressively harder. Last, the Digit-Symbol test involves speeded writing of symbols that correspond to numbers provided in a legend at the top of the page.

Serving as controls, Chelsea’s parents and two of her sisters were also administered the WAIS-R and Raven’s Matrices at a single time point that occurred between Chelsea’s 2nd and 3rd testing sessions. Her family was administered the Raven’s Standard Progressive Matrices, a more advanced version for adults whereas Chelsea was administered a simpler version, the Raven’s Coloured Progessive Matrices, which is administered to children. The data described below for all testing are presented as percentiles based on age-corrected, published norms.

### Results

**FIGURE 4:**
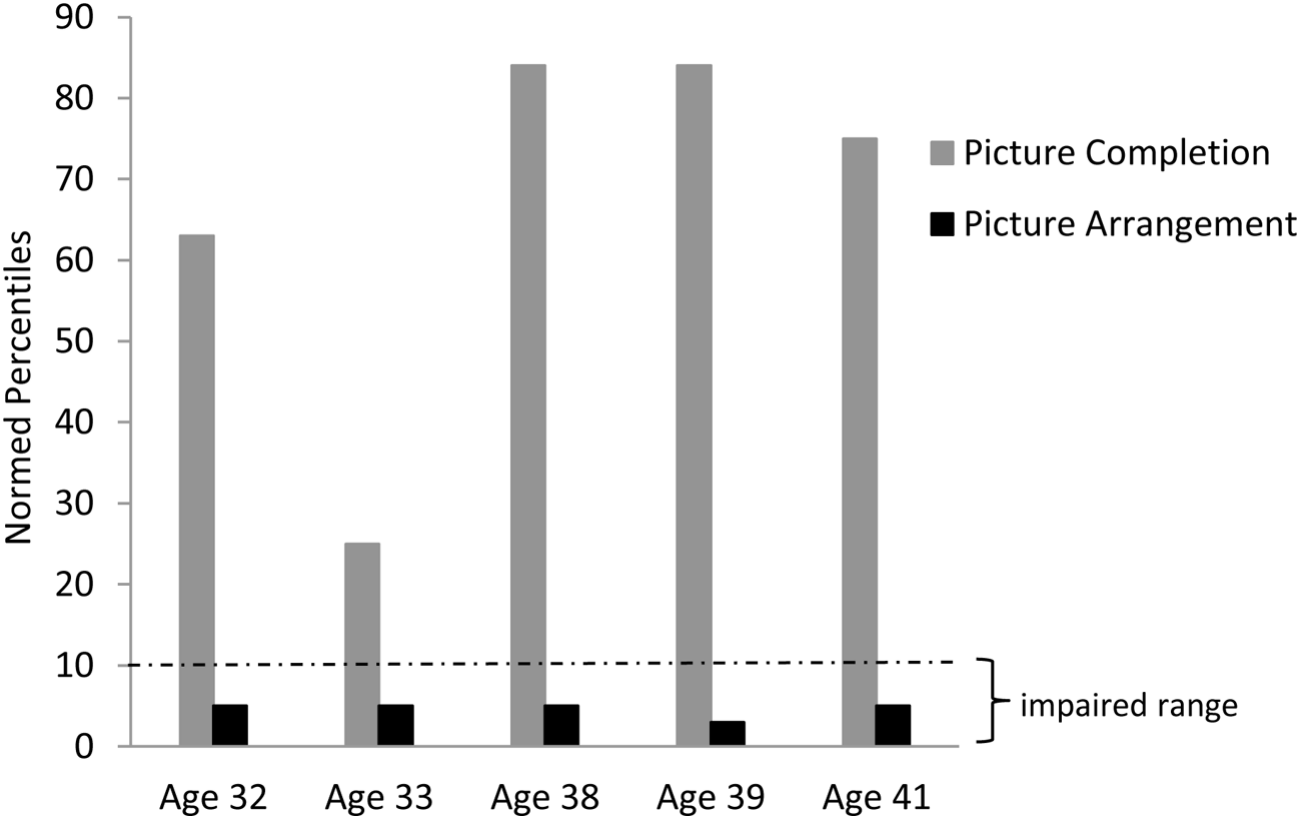
**Chelsea’s discrepant performance on the WAIS-R Picture Completion vs. Picture Arrangement tasks across several different testing sessions**.

Consistent with our findings in aphasic individuals, Chelsea showed a large discrepancy in performance between the Picture Completion and Picture Arrangement subtests of the WAIS-R. This discrepancy is even more striking in Chelsea than in the aphasic individuals: her performance on the Picture Completion task ranged from the average to high average range across five different administrations, while her performance on the Picture Arrangement task was consistently in the impaired range (based on age-adjusted WAIS-R norms; see **Figure 4**). Importantly, she was able to solve the first item on the Picture Arrangement task, indicating that she understood the task instructions. Furthermore, her poor performance on the Picture Arrangement task was not explained by exceeding time limits on the task, as she rearranged the cards (incorrectly) with time to spare. In contrast to Chelsea’s performance, her parents’ and two sisters’ scores were all in the average to high average range on both of these tasks.

Chelsea performed in the average to high average range on two other non-verbal WAIS-R subtests that are less dependent on reasoning, Block Design and Object Assembly. Like the Picture Arrangement and Completion tasks, these subtests also require visual perceptual processing and attention as well as a manual response. On the other non-verbal subtest of the WAIS-R, the Digit-Symbol test, Chelsea initially performed in the impaired range as it requires speeded writing with a pencil, to which she was not accustomed, but by the last administration, she performed in the average range. This striking contrast in Chelsea’s performance across WAIS-R subtests lends further support to the idea that language competence is related to reasoning performance; if Chelsea’s poor performance were due to her lack of formal education or some other general cognitive impairment, one would expect to see consistently impaired performance across all WAIS subtests.

Note that Chelsea’s scores on the first two administrations are difficult to see since they approach the x-axis at 0. The normal cut-off at the 10th percentile is shown.

**FIGURE 5:**
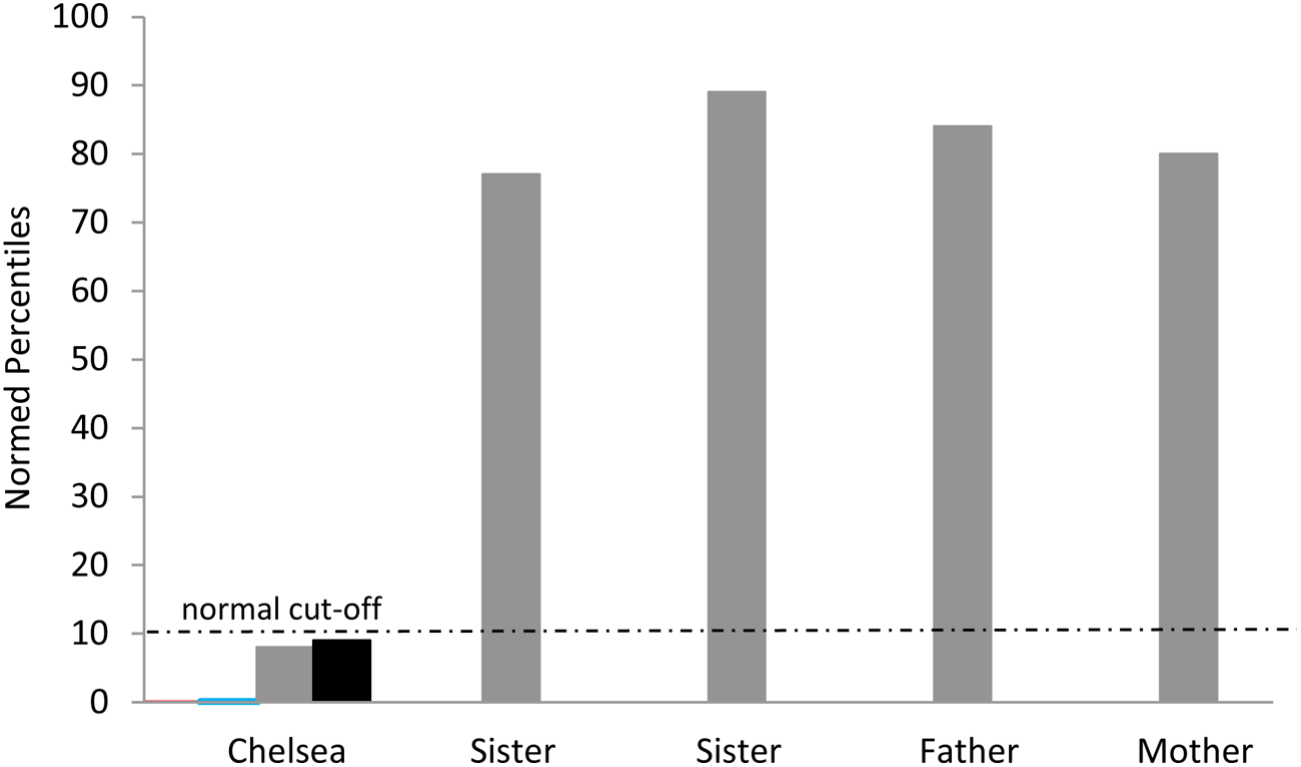
Chelsea’s performance on Raven’s Coloured Progressive Matrices at four different time points (normed percentiles ranged from <0.01 -9th percentile) and her family’s performance on a single administration of Raven’s Standard Matrices.

Chelsea was also tested on the Raven’s Coloured Progressive Matrices at four different time-points. Similar to findings in the aphasic individuals described above, Chelsea performed in the impaired range on this test. **Figure 5** shows her performance in relation to that of her two sisters and parents who all performed in the normal range based on normative percentiles. Importantly, just as with our findings in aphasic individuals described above, Chelsea showed a dissociation across different types of Raven’s items, correctly solving 100% of items requiring visual-pattern completion but only 20% of those requiring relational reasoning.

In summary, Chelsea, a congenitally deaf individual with poor language skills, showed disproportionately impaired performance on standardized neuropsychological tasks that involved reasoning. Based on her pattern of performance as well as a comparison with other family members who also had limited educational backgrounds, we conclude that her impaired reasoning skills may be in part due to her restricted language abilities.

## Discussion

In this paper, we have argued that there is a close relationship between language competence and the ability to reason and problem-solve. We have come to this conclusion based on our own work and the work of others showing that individuals with impaired language, particularly those with core language deficits (i.e., impaired comprehension and disordered language output), exhibit diminished performance on tests of reasoning and problem-solving (Piercy, 1964; De Renzi et al., 1966; Borod et al., 1982; Larrabee, 1986; Hjelmquist, 1989; Hamsher, 1991; Baldo et al., 2005, 2010). Specifically, we described data from a series of studies in which we compared the performance of aphasic (i.e., language-impaired) and non-aphasic stroke patients on a variety of reasoning and problem-solving tasks, such as the WCST and non-verbal subtests of the WAIS. In these analyses, the aphasic group as a whole was disproportionately impaired on reasoning tasks relative to the non-aphasic group, but the two groups showed comparable performance on other cognitively demanding tasks that did not involve reasoning. In the second part of the paper, we described complementary findings from a case of a healthy individual with delayed exposure to language due to an unaddressed congenital deafness. She, too, exhibited marked impairment on tasks of reasoning that stood in stark contrast to her ability to perform in the average to high average range on numerous cognitive tasks that did not involve reasoning. Taken together, these data are highly suggestive of an important role for language in reasoning and problem-solving.

Our findings in language-impaired individuals are consistent with previous studies in non-human animals and children that also suggest an association between language and reasoning. With respect to animal research, studies have shown that language-training in non-human primates facilitates problem-solving performance (Premack, 1983, 2007). Thompson et al. (1997) further showed that this facilitation is likely due to the learned ability of these animals to associate a token with an abstract relation, much like language provides us with words that can singly represent other propositional knowledge. In studies with children, it has been shown that the level of language competence and the use of private speech are directly related to problem-solving performance (Berk and Garvin, 1984; Berk, 1986; Winsler et al., 1997; Hermer-Vazquez et al., 1999; Fernyhough and Fradley, 2005; Carpendale et al., 2009). Vygotsky (1978, 2012) championed this idea that language plays a role in children’s development of reasoning skills: initially overt speech and dialoguing with elders is used to work through problems and is later internalized and becomes covert or inner speech. Before him, Piaget (1967) believed that language, while not critical for most stages of development, did play a role in formal operations when abstract reasoning emerges. In support of this idea, performance on Piagetian tasks involving formal operations is impaired in language-delayed individuals, while performance on tasks involving concrete operations is relatively intact (Furth, 1966; Furth and Youniss, 1971; Twilling, 1984). This dissociation between concrete and formal operations was also exhibited by Chelsea, the deaf individual with language impairment described above.

If we accept the idea that language facilitates problem-solving and reasoning in some way, this still leaves the question: what is the mechanism underlying this relationship? The answer to this question remains elusive and was not the focus of our investigations reported above, but a number of data points are instructive. First, prior work from our group and others have shown that articulatory suppression in healthy individuals (e.g., vocalizing nonsense syllables or irrelevant speech while doing a task) is disruptive to performance on reasoning/problem-solving tasks, suggesting that some form of verbal mediation (e.g., talking to oneself) facilitates reasoning and problem-solving (Hermer-Vazquez et al., 1999; Baldo et al., 2005; Wallace et al., 2009; Lidstone et al., 2010; but see Learmonth et al., 2008; Bek et al., 2010; Forgeot d’Arc and Ramus, 2011). Importantly, control conditions with non-verbal distraction (e.g., tapping a rhythm) are much less disruptive, showing that the effect is specific to verbal disruption, not a general disruption of attention or some other process. Furthermore, as in children, it has been shown that when healthy adults think out loud on a reasoning task, performance can improve (see Fox and Charness, 2010). Such studies in healthy individuals provide a causal link between language and reasoning.

In keeping with this idea, Sokolov (1968/1972) described studies in which individuals doing mental arithmetic and repeating words had recordable muscle activity in the articulators (e.g., tongue, lips, etc.), suggesting that such inner speech is literally that—covert vocalization. More recent studies measuring muscular activity show incredible specificity: when participants silently read the letter “P,” muscular activity was detected in their lips and when they silently read the letter “T,” muscular activity was detected in their tongue (McGuigan and Dollins, 1989; but see discussion below regarding dissociations of covert and overt speech). Sokolov concluded: “Inner speech emerges as a rather intricate phenomenon, where thought and language are bound in a single, indissoluble complex acting as the speech mechanism of thinking” (p. 1; also see Clark, 2006). Similarly, Carruthers (2002) echoes this notion: “Central cognition may also deploy the resources of the language system to generate representations of natural language sentences (in “inner speech”), which can similarly be of use in a variety of conceptual reasoning tasks” (p. 658).

A more systematically studied concept that can be invoked to explain the role of verbal mediation or inner speech is verbal working memory (Baddeley and Logie, 1999; Baddeley, 2000; Al-Namlah et al., 2006; Marvel and Desmond, 2012; Perrone-Bertolotti et al., 2014). It may be that this mechanism underlies successful problem-solving performance, as it provides a real-time rehearsal and updating of relevant information that can provide cognitive flexibility on reasoning and problem-solving tasks (Jonides, 2000; Emerson and Miyake, 2003; Carpendale et al., 2009). Verbal working memory may also facilitate problem-solving/reasoning by focusing attention and supporting self-cueing and self-monitoring (Clark, 2005; Unsworth and Engle, 2007; Forgeot d’Arc and Ramus, 2011). Verbal working memory, as measured by tasks such as repetition, is impaired in many aphasic individuals (Brown, 1975; Goodglass, 1992; Kohler et al., 1998; Baldo et al., 2012), and we have shown that patients’ repetition scores correlate with the percent of perseverative errors on the WCST and performance on Raven’s Matrices (Baldo et al., 2005, 2010).

In our current findings described above, repetition impairment, along with comprehension impairment, was related to poor performance on the Picture Arrangement (reasoning) task, although comprehension alone was most strongly related to performance on the WCST. Moreover, we also found that individuals with transcortical sensory aphasia (who have impaired comprehension but relatively preserved repetition) were greatly impaired in their reasoning performance, suggesting that core language (rather than simple repetition) is most strongly related to reasoning performance. By “core language,” we refer to the ability to both formulate and comprehend meaningful language. That is, while posterior patients with Wernicke’s/transcortical sensory aphasia (and global aphasics as well) are defined in part based on their poor comprehension, they also have a corresponding inability to produce meaningful language. Presumably, this limited ability to produce meaningful, overt language is paralleled by a limited ability to produce meaningful inner speech, a supposition which has only just recently begun to be tested more systematically (see Fama et al., 2014; Hayward et al., 2014, discussed below). Our speculation is that disordered inner speech is central to the reasoning performance decrements we observe in many language-impaired individuals. Such suppositions need to be explored in future studies to assess the extent to which inner speech is disordered in different aphasic subgroups and demonstrate how this relates to impaired reasoning (Kinsbourne, 2000).

Interesting insights on the role of inner speech in reasoning come from Jill Bolte Taylor, the neuroanatomist who suffered a left hemisphere stroke and later recounted her subjective experiences (Taylor, 2008; Morin, 2009). Taylor describes a striking loss of inner speech that accompanied her aphasia and negatively impacted her ability to reason and think through problems:

*The most notable difference between my pre- and post-stroke cognitive experience was the dramatic silence that had taken residency inside my head. I just didn’t think in the same way. Communication with the external world was out. Language with linear processing was out. But thinking in pictures was in* (pp. 75–76).

A similar parallel between overt and covert language loss in aphasia has also been described in other case studies of severe aphasia (Lecours and Joanette, 1980; Kertesz, 1988).

In contrast, individuals whose aphasia is more related to production deficits (e.g., Broca’s aphasia, anomic aphasia) appear to retain some capacity to generate inner speech, which might explain their residual reasoning ability. Although difficult to study, evidence for this capacity comes from two recent studies that assessed inner speech and the tip-of-the-tongue phenomenon in a group of aphasic individuals who had primarily output production deficits (Fama et al., 2014; Hayward et al., 2014). In Fama et al. (2014), 82% of the aphasic individuals reported hearing the words they wanted in their head but being unable to articulate them. Both Fama et al. (2014) and Hayward et al. (2014) reported anatomical and functional dissociations that mirrored the patients’ self-reports, such as fMRI activity in brain regions associated with phonological access (in left superior temporal cortex) when patients had this subjective experience. Single cases with such a dissociation between inner speech and overt language capacity have also been reported (Lecours and Joanette, 1980; Hanley and McDonnell, 1997).

Findings from the current study stand in seeming contrast to a handful of smaller case studies that have concluded that language is not critical for reasoning. For example, Varley and Siegal (2000) reported a case study of an individual with agrammatic aphasia who was trained to understand and then pass a theory of mind (perspective-taking) test. Another study by this group (Bek et al., 2010) tested five individuals with aphasia on a spatial-landmark conjunction task used previously to show the reliance of such tasks on language (Hermer-Vazquez et al., 1999). They found that language was not critical for performance, but they also concluded that such tasks are likely “assisted by” language (p. 656), consistent with our position. Last, Apperly et al. (2006) showed that a severely aphasic individual was able to pass first- and second-order false belief (theory of mind) tasks, again concluding that grammar is not a requisite for such performance.

One likely explanation for differing conclusions about the role of language in reasoning between our work and others’ is a difference in the types of patients investigated. What we refer to as “severe aphasia” is a syndrome in which patients have core language deficits: they cannot generate meaningful language, they are far below chance on simple word-picture matching tasks, and they have difficulty understanding simple sentences. In the case studies described above, the use of the term “severe aphasia” refers to primarily agrammatic patients who are well above chance on basic language tasks such as single word-picture matching vs. our severely aphasic patients who score in the very impaired range on such tasks. Our studies also include individuals with severe agrammatism, and these individuals do relatively well on our reasoning tasks. We believe they do well because their core language is less impaired and they may thus possess relatively preserved inner speech (see discussion of overt vs. covert speech above). In our opinion, the individuals who provide the best test of the role of language in reasoning are patients with severe, core language impairments who have an inability to generate meaningful language (e.g., individuals with chronic Wernicke’s aphasia) and who likely have disordered inner speech. These types of patients are more rare (relative to agrammatic patients), and it has taken many years to be able to analyze data from a group of such individuals.

Another explanation for the different conclusions reached by previous case studies of language and reasoning is the types of tasks employed. Most of these previous case studies focused on theory of mind/perspective-taking tasks, whereas we have focused on standardized neuropsychological tasks of reasoning and problem-solving like the WCST. Interestingly, in Apperly et al. (2006), the individual with severe aphasia who was able to perform false belief tasks was greatly impaired on executive functioning tests that included the WCST. Similarly, Varley (1998) reported impaired performance on the WAIS Picture Arrangement (reasoning) task in a fluent aphasic patient who was able to pass theory of mind tasks. These dissociations suggest that the two types of tasks likely tap distinct functions. False-belief tasks, including theory of mind, do not require the same degree of planning, self-monitoring, and online processing as the standardized neuropsychological tasks used to test reasoning and problem-solving in our studies described above. It is perhaps for this reason that performance on these latter types of tasks is more strongly related to language ability.

At the same time, a number of other studies on theory of mind in children and in delayed language learners have shown that performance is related to language competence (de Villiers and Pyers, 2002; Hale and Tager-Flusberg, 2003; Schick et al., 2007; Pyers and Senghas, 2009; see de Villiers, 2007; Carpendale et al., 2009 for reviews). In an interesting experimental paper, theory of mind performance was shown to be disrupted in adults under conditions of articulatory suppression (verbal shadowing) but not tapping (Newton and de Villiers, 2007). However, there is debate as to the precise nature of the role that language competence plays in theory of mind tasks (de Villiers, 2007). Although we have not collected data on theory of mind tasks with our aphasic patients, our case study described above, Chelsea, was tested on a spatial perspective-taking task that involved minimal verbal instructions and required a simple pointing response. She was able to understand the task and comply with instructions when asked to point to the picture that matched the visual scene in front of her. However, she failed when asked to point to the picture that matched the scene in front of the examiner (who sat to her side). Given her excellent spatial skills on tasks such as the WAIS Block Design and Object Assembly subtests, this poor performance was more likely due to her inability to take another’s perspective but could also have been due to her impaired language-understanding. A similar problem arises when attempting to test aphasic individuals on theory of mind tasks: the instructions themselves necessitate a minimal level of language competence, even when the task is visually presented. To the extent that a patient can understand the instructions on a theory of mind task, even a visually presented one, likely indicates that their language is not completely impaired. Indeed, it would be impossible for us to successfully convey instructions for theory of mind tasks to the severely aphasic individuals that were most impaired on our reasoning tasks described above.

To ensure that we did not overlook any “exceptional” cases in our large datasets, we inspected our data for single individuals demonstrating a dissociation between core language and reasoning, that is, individuals with a severe impairment in comprehension/lexical-semantics such as those with Wernicke’s aphasia who nonetheless showed preserved reasoning. On the WCST problem-solving task, 70% of patients were able to sort between 2 and 6 categories (out of a possible 6) and not a single patient with severe language impairment was among this group. All of the individuals with Wernicke’s or transcortical sensory aphasia (i.e., patients with severe core language impairments) sorted 0–1 categories. On the WAIS Picture Arrangement task, 9 of the top 10 performers were non-aphasic (within normal limits on the WAB language battery), with the 10th patient being an individual with mild anomic aphasia. There was one individual with Wernicke’s aphasia who scored 70% correct on the task (moderately impaired), and the other three individuals with Wernicke’s aphasia were in the bottom 10 performers. In contrast, many of the Broca’s/agrammatic individuals in our study were able to perform well on both the WCST and Picture Arrangement tasks (exceptions were those Broca’s/agrammatic individuals with more severe comprehension deficits), similar to the previous case studies of agrammatic aphasia. Thus, we would concur with previous studies suggesting an independence of *grammar* and reasoning (e.g., Varley and Siegal, 2000), but suggest that core language processes such as those most typically affected in Wernicke’s aphasia play an important role in reasoning. As suggested above, further work is needed to further explore these dissociations among different aphasic subgroups.

It should be noted that we are not claiming here that intelligence, or thought more generally, is necessarily dependent on language (also see Carruthers, 2012). Rather, we restrict our claims to higher-level reasoning/problem-solving, that is, the kind of thought normally facilitated by verbal mediation or inner speech (Sokolov, 1968/1972; Carruthers, 2002; Evans, 2008). This distinction has been made by Evans (2008) who contrasts a heuristic, *quick and dirty* system that “rapidly contextualize[s] problems with prior knowledge and belief” vs. a slow and serial system that is engaged during “conscious effortful analytic reasoning” (p. 261). He argues that the former is a non-verbal system that we share with other animals, while the latter system is dependent on language and is unique to humans.

Nor are we making the claim that language is absolutely indispensable to reasoning—we have rather argued throughout this paper that language can “facilitate” and is “supportive” of higher-level reasoning capacity. Even Ratliff and Newcombe (2008) who were skeptical of the original Hermer-Vazquez et al. (1999) shadowing study on reasoning and language reported that the original findings hold up to a certain degree. They concluded that, although language may not be “crucial” to reasoning, it is “helpful.” Similarly, Forgeot d’Arc and Ramus (2011) showed that verbal shadowing impaired performance overall on a non-verbal task involving belief attribution but that performance was still above chance. Although they felt their data supported the idea that belief attribution is independent from language, they also concluded that language acts as “an additional tool to keep relevant information in short-term memory” (p. 984). Similarly, Varley (2014) concluded that “language resources may often be deployed to scaffold performance on a range of problems” by way of supporting short- and long-term memory (p. 242). Again, this is consistent with the idea that language can serve a facilitatory role in online processes that support reasoning performance.

Finally, we are not suggesting that the use of inner speech and language to support reasoning is somehow predetermined; it is apparently a learned phenomenon (Vygotsky, 2012) that can vary across individuals. Interesting cross-cultural research has also shown that the use of inner speech, while facilitatory for non-verbal reasoning in European–Americans (healthy individuals), can be disruptive for non-verbal reasoning in East Asian–American participants on particular tasks (Kim, 2002). This finding makes the intriguing prediction that reasoning performance in East Asian patients with aphasia would not show a similar pattern of disruption as in our current study which included predominantly European–American patients. We are currently investigating this prediction with a cross-linguistic study of aphasia in collaboration with colleagues in Taiwan. It is likely that the degree to which language is invoked to support reasoning within and across cultures depends on the type of task involved (e.g., supportive strategies for particular tasks in one culture may be more visual-spatial while in another, more verbal). More experimental work in this intriguing area of investigation is clearly needed.

Brain imaging studies have also provided novel insights into the relationship between language/inner speech and cognition (for a review, see Girbau, 2007; Morin and Hamper, 2012). Consistent with our findings, Pillay et al. (2014) found that posterior brain regions overlapping with Wernicke’s area, including the left posterior superior temporal gyrus and inferior parietal cortex, were most closely related to pre-articulatory phonological access (what they and others have used as a stand-in for inner speech). However, some recent functional imaging studies in healthy individuals have suggested that language areas can dissociate from higher-level cognitive processes (Monti et al., 2007; Willems et al., 2010; Fedorenko et al., 2011). In our work, we have shown that lesions involving language areas in the left hemisphere (most consistently, left posterior temporal cortex) are associated with decreased performance on reasoning and problem-solving tasks (Baldo et al., 2005, 2010). In Baldo et al. (2010), we found a dissociation between brain regions associated with performance on the visual-pattern completion items on Raven’s Matrices vs. performance on the relational reasoning items: the former was associated with visual association regions in left inferior temporo-occipital cortex and the latter, with left posterior middle and superior temporal cortex (a critical language zone). Still, caution is warranted in drawing strong conclusions from lesion data as there is also the possibility that distinct but overlapping networks underlie language and reasoning processes in the brain (Kertesz, 1988; Baldo et al., 2010).

In short, a range of philosophical inquiries and experimental evidence supports the idea that language and reasoning are highly inter-dependent. Further experimental investigations of this relationship are needed in order to establish more evidence for a causal role of language in reasoning (Varley, 2014), especially with respect to the putative role that inner speech plays as a mediating mechanism. We conclude here with an elegant description by O’Brien and Opie (2002) of the relationship between language and cognition:

*Natural language thereby becomes a powerful cognitive tool… one that can regulate the sequencing of thought, via the constant interplay between networks that encode linguistic signals and those that encode thoughts… Such causal loops catch up language and thought in a tight web of mutual influence that extends our cognitive capacities well beyond those of infra-verbal organisms* (p. 327).

## Conflict of Interest Statement

The authors declare that the research was conducted in the absence of any commercial or financial relationships that could be construed as a potential conflict of interest.
